# Surgeons’ Perceptions on the Utility of a Conceptual Novel Force Sensor at the Surgeon-Tool Interface: Formative Interview Study

**DOI:** 10.2196/78845

**Published:** 2026-04-27

**Authors:** Bennet Mühlenbeck, Jeremy Opie, Carmen Salvadores Fernandez, Shireen Jaufuraully, Dawn Parris, Adrien Desjardins, Anna L David, Dimitrios Siassakos, Manish K Tiwari, Ann Blandford

**Affiliations:** 1 UCL Interaction Centre (UCLIC), University College London, London, United Kingdom; 2 UCL Hawkes Institute, University College London, Gower Street, London, WC1E 6BT, United Kingdom, 44 0207679 ext 9575; 3 Nanoengineered Systems Laboratory, Mechanical Engineering, University College London, London, United Kingdom; 4 Institute for Women’s Health, University College London, London, United Kingdom; 5 Department of Electrical and Computer Engineering, University of British Columbia, Vancouver, BC, Canada; 6 National Institute for Health and Care Research University College London Hospitals Biomedical Research Centre, University College London, London, United Kingdom

**Keywords:** haptics in surgery, force feedback, sensorized glove, user requirements, surgeon-tool interface

## Abstract

**Background:**

Real-time force feedback is essential in many surgical specialties. While previous research has focused on force measured at the tool-tissue interface, little work has explored the benefits, limitations, or opportunities of measuring force at the surgeon-tool interface.

**Objective:**

This study aims to explore scenarios in which surgeons from different medical specialties and experience levels could benefit from receiving feedback on the force exerted at the surgeon-tool (or surgeon-tissue) interface.

**Methods:**

Exploratory qualitative research was conducted through interviews with medical practitioners (N=15). This study explored perceptions of a conceptual novel force-sensing surgical glove that could provide real-time feedback in terms of usability, utility, value, and limitations. Opportunities and barriers to implement a sensor of this type in clinical practice were also explored. Participants had experience in anesthetics, dental surgery, plastic and dermatological surgery, general surgery, and obstetrics and gynecology, as these surgical fields all require precise feedback on exerted forces.

**Results:**

Participants identified two key areas where a force sensor could yield significant benefits: (1) it could enhance surgical training through objective skill assessment and quantifiable feedback, and (2) it could provide valuable insights into the forces applied during practice, particularly in scenarios where other sensory feedback is masked. Participants appreciated that a sensorized glove that can provide real-time force sensing at the surgeon-tool interface would allow for continued feedback irrespective of the instrument, and integrate seamlessly into their current surgical workflow. Furthermore, as surgeons in some specialisms, for example, dental or obstetrics and gynecology, perform manual tasks, having a sensorized glove would provide feedback in instances where they are physically manipulating tissue. However, participants expressed concerns about accurately defining safe force ranges due to the variability in patients’ anatomical structures and the potential interference with tactile sensation.

**Conclusions:**

Surgeons from various clinical practices agreed that force sensing at the surgeon-tool interface could be valuable and provide them with optimal versatility as to when they would adopt force sensing. A sensorized glove could improve decision-making and surgical outcomes when other sources of information guiding force exertion are masked. Conversely, it could be detrimental when the organic information to guide force exertion is distorted when using the sensor. While the choice between interaction modalities is dependent on the accessibility of different senses during surgery, design suggestions as to where sensors are best placed on a sensorized glove are dependent on the instrument used or the type of manual procedure conducted.

## Introduction

When treating medical disorders operatively, surgeons must apply physical force to manipulate human body tissue [1]. Applying an appropriate amount of force is pivotal to the success of surgeries since excessive or insufficient force application can damage bodily tissue or prevent task completion [2]. Nonetheless, applying appropriate force levels poses a significant challenge for novice and expert surgeons [3,4]. Thus, surgeons rely on real-time feedback to determine what force to exert depending on the type of surgery [5,6].

The importance of real-time force measurement in surgery has long been acknowledged and has been deemed desirable in many medical specialties, including plastic surgery [7], anesthetics [8], dentistry [9], and obstetrics and gynecology [10], demonstrating the importance of force sensing across medical specialties.

In recent years, scientists have investigated the usability of sensors attached to diverse surgical tools that quantify the pressure surgeons apply to their patients’ tissues [2,3,11,12]. Such force-sensing devices’ applicability is limited by issues around biocompatibility and sterilization, long-term stability, and adaptation to surgical tools [13]. In response, researchers have developed novel surgical tools with smart force-sensing abilities [14]. However, such force-sensing systems only enable force measurement for individual surgical instruments [15]. This presents high costs, and inconsistencies between instruments’ force sensing could distract operators; for example, each instrument may use a different method of visualizing and reporting force measurements [13].

**Figure 1.**
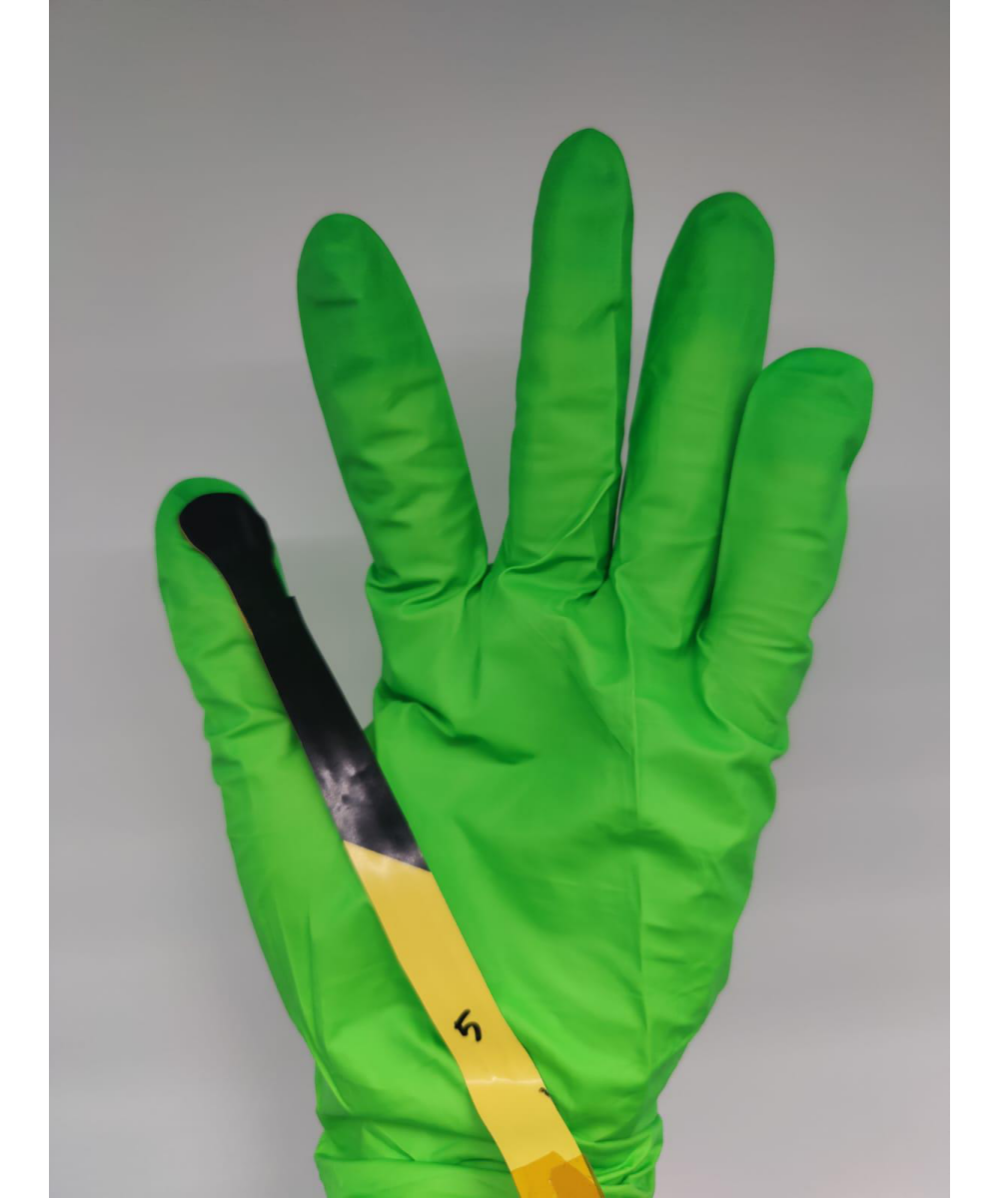
An example of a novel force sensor surgical glove prototype.

Layard Horsfall et al [16] developed a piezoresistive sensor that can be attached to the fingertips of surgical gloves, similar to that in Figure 1. They aim to address the limitations of existing intraoperative force measurement methods and enable a practical, effective, and affordable method to measure intraoperative forces [17]. Additionally, the glove is designed for sterility and provides real-time feedback of the force being applied to instruments. To evaluate the glove, Layard Horsfall et al [16] assessed expert and novice surgeons’ force exertion during a grape dissection task, which is a common surrogate task performed by neurosurgeons. When mounted on a surgical glove, they found that the sensor could detect statistically significant differences in the amount of force expert and novice surgeons applied to a scalpel during the task, revealing significant potential for improvement in surgeons’ application of intraoperative force. However, the magnitude of intraoperative forces required during surgery varies significantly between and within surgical specialties depending on the maneuver performed, the tissue manipulated, and the instrument used [1]. Therefore, for a novel sensor to be beneficial to specific surgical specialties, it is important to identify which procedures within those specialties force feedback would offer the most value. Based on this information, a sensor’s feedback can be customized to account for the variations of intraoperative forces applied across and within surgical specialties.

Existing literature proposes that measuring manually applied forces carries benefits across multiple areas, including surgical practice, training, and assessment of junior staff.

Most surgical procedures require the exertion of light touch. For example, the analysis of error types by Tang et al [18] made by surgical trainees during laparoscopic cholecystectomies revealed that more than 50% of errors emanated from participants exerting excessive force. These findings led several studies to investigate how real-time feedback on forces applied to human tissue affects force exertion. Golahmadi et al [1] conducted a systematic review of 6 studies that compared the amount of force applied when feedback was provided versus when no feedback was provided, concluding that forces decreased when feedback systems existed. These results also indicate that real-time feedback on exerted forces could improve surgery outcomes.

Besides direct benefits on surgical outcomes and patient safety, force sensing can substantially improve the training of novice surgeons who currently learn through subjective feedback from their mentors [19,20]. As tolerance for operating errors decreases, the demand for accurate force exertion training methods is growing [21]. Previously, real-time feedback systems for manual force were found to optimize novice surgeons’ operating techniques. The impact of real-time force feedback during training on surgical trainees’ tissue-handling learning curve was assessed [22]. In a randomized controlled trial, 72 medical students conducted 6 different suturing tasks under real-time force feedback, postprocessing force feedback, or no force feedback (control). Students significantly improved tissue-handling performance when force feedback was present.

In addition to its role in surgical training, force sensing could provide a quantitative metric of surgical skill for objective assessment of the outcome quality of surgical tasks [23,24]. Cundy et al [20] developed a force-sensing box trainer for laparoscopic surgery and validated different force variables as indicators of surgical skill for 5 curriculum tasks. The overall magnitude of the force exerted during the task indicated the participants’ experience level, highlighting the suitability of force measurement as an assessment method.

For the force sensor to be applied during specific medical procedures, it must account for the variations of intraoperative forces between and within surgical specialties.

Golahmadi et al [1] reviewed studies conducted in various surgical specialties that measured forces exerted at the tool-tissue interface. They found significant differences in average and maximum forces applied between different surgical specialties when averaging across tasks and models. Average forces found in ophthalmology (0.04 N), neurosurgery (0.68 N), vascular surgery (0.07 N), and cardiothoracic surgery (1.47 N) were significantly smaller than those recorded in general surgery (4.67 N), ear, nose, and throat surgery (8.49 N), obstetrics and gynecology (8.69 N), and urology (9.79 N). These findings emphasize the need to investigate the potential value of a sensor glove in different surgical specialties.

The force applied during surgery largely depends on the specific maneuver medical practitioners perform. Sugiyama et al [25] investigated forces applied using bipolar forceps during frequently performed neurosurgical tasks to build a catalog of force profiles. They quantitatively compared forces exerted during different neurological tissues’ coagulation, grip, division, and dissection and found that force measurements differed statistically significantly between selected maneuvers with bipolar forceps. These findings highlighted the need to determine safe force ranges for surgical maneuvers.

Across surgical specialties, forces applied differ significantly due to the various manipulated tissues. Golahmadi et al [1] compared the mean average and the mean maximum force applied to the 4 main tissue types (nervous: n=17; epithelial: n=18; muscle: n=3; connective: n=10) and discovered significant differences between the intraoperative forces applied. Nervous tissue received the smallest mean average (0.4 N) and mean maximum (1.7 N) intraoperative force, followed by epithelial and muscle tissue (mean average: 3.8 N, 4.1 N; mean maximum: 9.7 N, 6.7 N), and connective tissue received the highest mean average (45.8 N) and mean maximum force (347.9 N). This variance of forces exerted across human body tissues highlighted the need to identify where the sensor could best be applied.

The forces required to manipulate human body tissue can also vastly differ inter- or intraindividually. Fernee et al [26] investigated the variation of dental tissues within and between 30 healthy individuals’ first incisor and second premolar teeth using Bayesian multilevel modeling and revealed that dental tissues differed in volume and surface area at the individual and tooth levels. Furthermore, Di Stasio et al [27] investigated normal values of epithelial thickness for 6 different anatomic locations in the oral cavity using a sample of 28 healthy patients, discovering that the SD of mean thickness exceeded 10% of the mean value for 5 of 6 individual anatomic locations.

Each surgical instrument demands specific magnitudes of force since the instrument’s handling and the force transfer to the tissue vary between tools. A review by Golahmadi et al [1] demonstrated that the intraoperative forces that surgeons apply differ according to how they use their instruments.

However, beyond the tool-tissue interface, there is also the surgeon-tool interface, where the sensor is placed on the surgeon to measure forces applied to the tool. Rosen et al [28] conducted a study exploring the forces applied at the surgeon-tool interface in teaching scenarios as an objective measurement of skill in minimally invasive surgery and found that experienced surgeons apply much less force and torque than novice surgeons, which was later corroborated by the study of Layard Horsfall et al [16]. Furthermore, Aggravi et al [29] compared intraoperative forces exerted at the surgeon-tool interface using bipolar forceps, a spatula, and a suction tool. They found that forces exerted by the thumb, index, and middle fingers differed for the different tasks conducted with the different instruments. These findings imply that the local spread of exerted force must be considered in creating real-time feedback for individual maneuvers. Nevertheless, some interventions, such as minimally invasive laparoscopic surgery, require force from surgeons in different ways than manual force [30]. However, few studies have explored force sensing at the surgeon-tool interface. Additionally, in some instances, surgeons may perform manual interactions with the patient, physically interacting with tissue, which is described as the surgeon-tissue interface. In this study, we are treating these as equivalent. Thus, from this point forward, when we discuss the surgeon-tool interface, we are also including the surgeon-tissue.

While most studies have investigated the use of intraoperative force through measurement at the tool-tissue interface, there is little work that has explored the benefits, limitations, or opportunities of measuring force at the surgeon-tool interface across and within different medical specialties. Thus, it is critical to investigate in which surgical scenarios operators from different medical specialties can benefit from real-time force measurement at the surgeon-tool interface.

This study aims to explore these scenarios in which surgeons from different medical specialties and experience levels could incorporate the use of a conceptual novel force sensor that would provide real-time feedback on the force exerted at the surgeon-tool interface.

To address this issue, we conducted a series of semistructured interviews. Our approach consisted of showing participants a concept video of a novel force sensor and generating conversation as to how such a technology could be adopted into their clinical practice. This work is a formative, exploratory investigation intended to generate initial insights into an underresearched area and inform future research and design iterations.

## Methods

This study comprised 30-minute-long semistructured interviews with surgeons from 5 different medical specialties. Semistructured interviews allowed us to elicit detailed narratives [31] and a succinct postinterview generalization of insights beyond the borders of surgical specializations.

### Ethical Considerations

Ethics approval for this study was granted by the local ethics committee (reference UCLIC_2022_002_Costanza). Informed consent was obtained from all participants, who were provided with an information sheet, and all data were anonymized to maintain participant privacy. Additionally, each participant received approximately US $25 Love2Shop (the United Kingdom) or approximately US $26 Amazon (Germany) voucher for contributing to this research.

### Participants

Through convenience sampling, 15 medical practitioners with operative expertise (male: n=9 and female: n=6) were recruited from specialties where force application is critical and varied (soft vs hard tissues and instrumental vs manual handling). Participants came from anesthetics (2/15, 13%), dental surgery (4/15, 27%), general surgery (3/15, 20%), plastic and dermatological surgery (3/15, 20%), and obstetrics and gynecology (3/15, 20%). At least 2 participants were sampled from each clinical background to ensure a balance between representing a broad range of medical specialties and enabling aggregation of interview results. Participants were recruited from Germany (9/15, 60%) and the United Kingdom (6/15, 40%). The only exclusion criterion was participants’ clinical experience, which had to be at least 1 year in practice and not exceed 2 years out of practice (mean 16.5, SD 12.4 years). Experience was recorded for descriptive purposes only and was not used as a proxy for surgical skill. Table 1 presents an overview of the participants.

**Table 1.** Overview of participant characteristics including specialty, sex, country, and years of experience within their surgical specialty.

ID	Specialty	Sex	Country	Experience (years)
AN1	Anesthesia	Male	Germany	25
AN2	Anesthesia	Male	United Kingdom	6
DS1	Dental surgery	Male	Germany	30
DS2	Dental surgery	Male	Germany	35
DS3	Dental surgery	Male	Germany	30
DS4	Dental surgery	Male	Germany	39
GS1	General surgery	Male	Germany	19
GS2	General surgery	Male	Germany	15
GS3	General surgery	Female	Germany	2
PD1	Plastic surgery	Male	Germany	10
PD2	Dermatological surgery	Female	United Kingdom	4
PD3	Plastic surgery	Female	United Kingdom	6
OG1	Obstetrics and gynecology	Female	United Kingdom	7
OG2	Obstetrics and gynecology	Female	United Kingdom	14
OG3	Obstetrics and gynecology	Female	United Kingdom	5

### Introductory Video for Concept Familiarization

**Figure 2.**
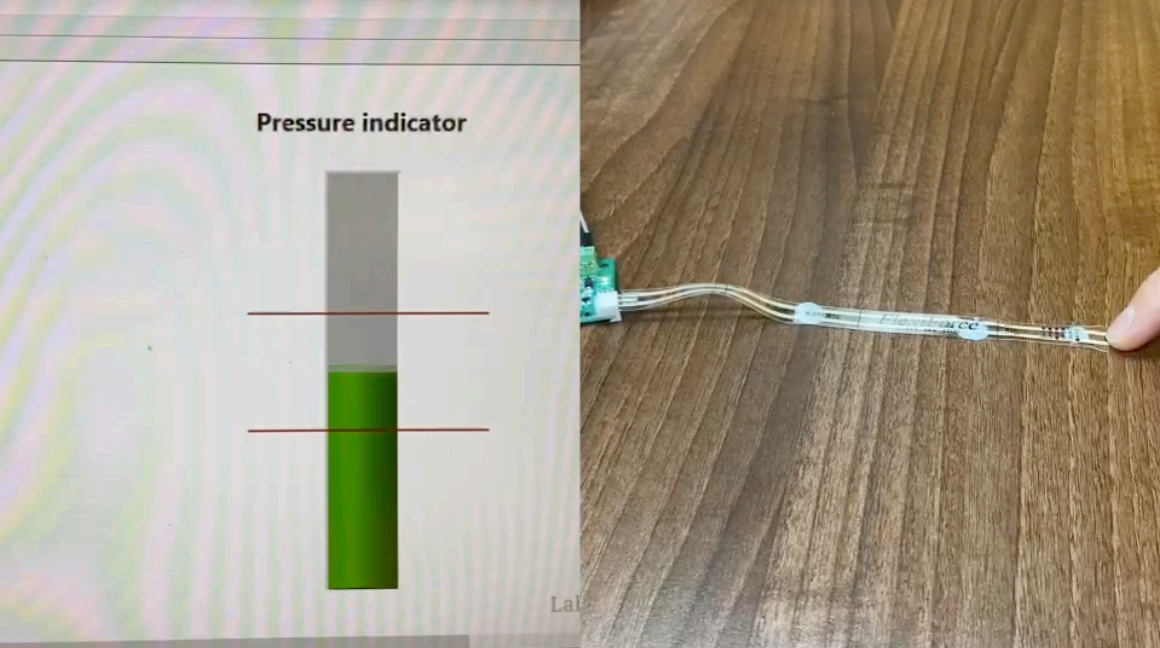
Video excerpt showing real-time feedback visual.

Participants did not interact with a physical prototype during the interview, as each surgical group would have required a different prototype relevant to their specialism, and this formative study sought to better understand user requirements for such prototypes. Therefore, feedback was based on a conceptual video demonstration of the novel force sensor. Each interview was introduced by showing a 2-minute video (Multimedia Appendix 1) to provide participants with a shared understanding of the concept rather than influence attitudes toward a specific implementation. The video contained spoken information on the sensor’s background, aim, functionality, and use in a clinical context and contained footage of the sensor applied to a surgical glove being used by neuroscientists in a grape dissection task provided by Layard Horsfall et al [16]. The video also showed moving images of the sensor being pressed alongside a real-time visualization of the force feedback (Figure 2). Two video versions were created, one with German and one with English audio, depending on the participant’s first language. After reviewing the video, participants were invited to pose questions about the functionality or use of the sensor glove.

### Study Design

The first 9 participants were asked to provide a detailed description of their most performed operative procedure prior to the interview to help the interviewer prepare. However, three of the first six participants did not provide this information, and requesting the information led to loss of contact with three further prospective participants, so this phase of the study was subsequently removed. The interviewer (who was not a medical professional) took steps to familiarize themselves with the relevant surgical procedures in preparation for each interview. For later participants, instead of discussing a predetermined procedure, they were asked to identify maneuvers in which applying appropriate force is crucial. This change did not appear to impact the results, as participants were still asked to discuss the potential use of the sensor within their daily practice, and it allowed participants to steer the discussion toward use cases they wanted to propose.

At the start of the interviews, surgeons participating online received an information sheet to read via email and a link to a digital consent form hosted on REDCap (Research Electronic Data Capture; Vanderbilt University) [32]. Those participating in person received a printed information sheet and signed their informed consent through REDCap on the interviewer’s laptop. Subsequently, participants were briefly reminded of the research goal and invited to ask questions about the study. All interviews were recorded using Microsoft Teams.

The semistructured interviews were conducted in person (n=1) or online using Microsoft Teams (n=14), depending on the participant’s preference, and lasted approximately 30 minutes to facilitate the recruitment of time-poor experts. As this was an exploratory study, no predefined end points were set; instead, interviews were designed to elicit broad insights into usability, opportunities, and barriers. In addition, as a formative investigation, no prototype was available for direct observation or usability testing. A semistructured interview guide (Multimedia Appendix 2) was used, containing open-ended and probing follow-up questions to gain in-depth insight into the medical practitioners’ perspectives on the potential use of a novel sensor. The interview comprised three phases: previous experience with force-sensing mechanisms, pressure-sensitive maneuvers, and potential benefits and limitations of a force sensor glove.

To begin, participants were asked to disclose their operative expertise and describe any experience with force-sensing mechanisms. Participants were then encouraged to identify challenges they faced during their work related to the exertion of manually applied forces. These questions aimed to help participants think about scenarios in which a novel force sensor could help them apply appropriate intraoperative force. The central part of the interview focused on pressure-sensitive maneuvers that they had identified before or during the opening phase of the interview. The interviewer prompted participants to think about how they might apply a novel sensor in their existing workflow and describe how it would affect their practice and added probing questions to understand participants’ motivation to use or not use a force sensor. Finally, participants were asked to consider the surgical glove’s benefits and limitations in the context of surgical training and assessment to determine whether a force sensor might have value in training junior surgeons. Additionally, participants were asked to consider the benefits and drawbacks of attaching a force sensor to the hand instead of having a sensor on the instrument and to debate the installation of different feedback modalities. Before finishing the interview, participants had the opportunity to make final remarks and ask any remaining questions.

### Data Analysis

The first author (BM) transcribed each interview using Microsoft Word and manually translated the transcripts of interviews held in German to allow for their analysis in English and better comparison across the dataset. The first author conducted a thematic analysis of the transcripts using NVivo (version 14.23.0; Lumivero). Initially, the transcripts were reviewed to establish a comprehensive understanding of their content. Initial codes were generated inductively, wherein meaningful segments relevant to the research aim were identified and labeled. These codes were then compared, grouped, and refined using affinity mapping to identify potential themes from the data. Subsequently, the identified themes were iteratively reviewed and validated with the original interview data to ensure their accuracy and relevance. Finally, the themes were refined. and named to create a comprehensive representation of the surgeons’ perceptions of usability requirements for a novel sensor. The analysis was inductive; thus, no predefined codes were used, and measures such as theoretical saturation, which presuppose a postpositivist approach to analysis, are inappropriate [33,34].

## Results

### Overview

**Figure 3.**
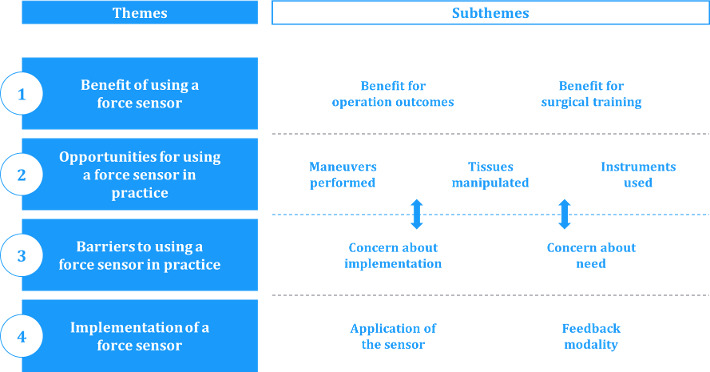
Overview of themes and subthemes.

In total, four top-level themes were identified: benefits, opportunities, barriers, and deployment of a force sensor (Figure 3).

### Theme 1: Benefits of Using a Force Sensor

All participants identified a range of ways in which a novel force sensor could benefit their work. The main anticipated benefits were its improvement of operative outcomes and the training of prospective surgeons.

#### Benefit for Operation Outcomes

Participants across different medical specialties expected that a novel sensor would enhance operative outcomes by offering real-time feedback on the surgeons’ exerted force. All participants identified scenarios within their disciplines where the application of inappropriate force causes harm to patients or prevents task completion. Therefore, all participants predicted that real-time feedback about their exerted force could increase patient safety within their operating environment: “I think if you know exactly how much force you’re applying, that may make you rethink what manoeuvres you’re doing to deliver that baby” (OG2).

Participants agreed that providing real-time feedback on exerted force using a sensor could positively affect patients’ health. Beyond patient safety, several participants indicated that the provision of force feedback could enhance their operation speed. During specific procedures, practitioners must carefully approach the correct magnitude of force; suitable force feedback would enable them to skip time-expensive approaches to reach adequate force levels. This would improve efficiency and effectiveness and could also create financial gains:

I have to decide whether I can sit down and work on it very slowly and carefully for half an hour, which is of course completely uneconomical. Or I just apply the appropriate force and it goes faster.

[DS2]

While the feedback could increase surgeons’ effectiveness and efficiency, it could also create a psychological benefit, which enhances their operations’ outcomes. For example, many surgeons reported that receiving information about how much force they are applying would boost their comfort and provide assurance about their performance: “It is a problem in today’s increasingly justiciable times that we still rely on feelings” (AN1). Furthermore, participants reported that their increased confidence could impact their operative performance positively. Finally, the indirect force feedback could also benefit operative outcomes in terms of patients’ psychological well-being during surgery. That is, feedback on the surgeons’ exerted force was proposed to comfort patients who are not under anesthesia: “It can be a tool for trust building in scenarios where women have been previously sexually assaulted or had very traumatic internal examinations” (OG3).

In summary, the interviews demonstrated that surgeons could profit from using a novel force sensor during practice to apply appropriate magnitudes of force, which could enhance operative effectiveness and efficiency.

#### Benefit for Surgical Training

Offering real-time feedback on surgeons’ exerted force through the use of a novel sensor was identified to benefit the training of junior doctors, as existing medical training methods are inappropriate and unstructured. Participants reported that, in most cases, junior doctors learn to perform surgery through “learning by doing” (GS1). Unsurprisingly, the current shortcomings of surgical training for junior doctors were judged problematic by participants who feared the outcomes of untrained surgeons’ mistakes:

And of course, you don’t want to endanger a patient just so that the new generation learns something. [...] but that’s the truth [...] at the moment.

[GS2]

Furthermore, the interviewees criticized that, where surgical trainees are learning in practice, the present teaching style is dominated by the apprentice model, whereby senior surgeons instruct junior surgeons through qualitative feedback, which is exceptionally challenging for trainees due to individual differences between trainers and trainees. Most participants agreed that there is a demand for objective feedback about their exerted force. Thus, the prospect of a force sensor that generates real-time feedback on forces elicited enthusiasm for its implementation in training inside operating theaters:

That’s where I see the advantage because they are still a bit lost, they don’t really have any benchmarks and they have to gain experience, which may be unnecessary, and patients have to suffer. So yes, maybe you can improve that with such a pressure sensor.

[DS3]

While many participants acknowledged the benefit of the sensor in practical training, others proposed that the sensor could be used in simulation training. According to the interviewees, existing methods and models for simulation training are unavailable, unsuitable, or unaffordable. Two-thirds of all participants expressed that a novel force sensor could demonstrate high usability in simulation training outside the operating theater. The main benefit would be that junior surgeons could develop their tactile senses and learn how much force is appropriate in specific situations before applying their skills to actual patients: “I think it would be an absolutely incredible tool because that way you could practice as much as you want before you apply it to a human being and potentially sever that facial nerve” (PD2).

Altogether, the interviews demonstrated that junior practitioners could profit most from a novel force sensor as a source of objective feedback on how much force they are and should be applying. Thus, they could use it to refine their skills outside and to guide their forces inside the operating theater.

### Theme 2: Opportunities for Using a Force Sensor in Practice

The second theme describes the opportunities to use a force sensor in practice. Participants rated the application of the sensor glove as meaningful depending on the characteristics of their maneuvers performed, tissues manipulated, or instruments used.

#### Maneuvers Performed

Across all medical specialties and experience levels, participants could imagine using a force sensor to assist them in various maneuvers in their work and identified maneuver-specific characteristics. Almost all participants expressed that they would appreciate an additional source of feedback in procedures where there is no external indication of how much force they are applying; thus, they must rely on their experience to determine appropriate magnitudes of pressure: “From my experience, it’s just based on what you’ve experienced previously because it’s a blind procedure, you can’t see what you’re doing” (OG1).

The interviews uncovered different reasons for surgeons’ limited awareness of their exerted force. For example, visual feedback from the surgical site can be obstructed or masked where sensitive tissue is hidden through other tissue or blood flow at the operating site: “They’re bleeding, and you can’t see whether you’re in the axial direction of the root” (DS4).

In some procedures, surgeons may be reliant on auditory feedback from patients to determine whether the force applied is appropriate. However, the use of anesthesia may result in application of inappropriate force not being apparent until after the anesthesia has worn off: “I can only say that if I don’t have any complications postoperatively, then obviously the procedure was fine, there’s not much more feedback there really” (DS3).

Thus, there is a significant opportunity to apply a force sensor during maneuvers where surgeons are unaware of their exerted force due to the absence of alternative sensory input such as direct visual or auditory patient feedback.

#### Tissues Manipulated

Procedures that require force sensing to avoid harm to the patient are usually defined by the practitioners’ interaction with particular bodily tissue. Participants frequently referenced the opportunity to apply a novel force sensor when dealing with tissue sensitive to excessive intraoperative force: “With nerves and vessels, you obviously have to be extremely careful because it depends on the size, but they’re usually quite delicate” (PD3).

Although most participants named fine, delicate tissues among those most sensitive to excessive pressure, one participant added: “When you’re required to apply quite larger forces for certain things, then it becomes quite important to know exactly how much” (PD3). The most force-sensitive tissue types are not necessarily the most delicate ones. Instead, participants labeled tissue as sensitive to pressure based on its importance for bodily functioning. The tissue types most frequently classified as critical for bodily functioning by participants across all disciplines were blood vessels and nerves:

The effect on damaging a nerve on the head and neck is quite profound. You’re talking about not being able to lift your eye [...] not being able to smile properly.

[PD2]

Additionally, participants pronounced that tissue types are sensitive to the amount of force applied when they have poor healing abilities. In that context, bones were frequently mentioned by general surgeons:

The thing with skin is [...], it will heal. But if you break a bone, it is a big deal.

[PD3]

Meanwhile, in dental surgery, connective tissue, which supplies other structures with blood, is deemed poorly healing: “If unnecessary pressure is applied to an instrument, this can lead to tissue tears, which are then difficult to manage” (DS1). Thus, the sensor was also considered to be useful when practitioners handle coarse tissue.

#### Instruments Used

In addition to force-sensitive tissues, those procedures that require force sensing to avoid causing harm to the patient are usually defined by the surgeons’ use of specific instruments: those that are susceptible to transmitting excessive force. Across medical specialties, participants reported using a variety of instruments that, when applied inappropriately, can cause significant harm to their patients and thus could benefit from real-time feedback: “If you clamp an artery too much, it’s broken” (GS2). However, one participant remarked that the potential to cause harm to the patient is not evident for all tools:

I think that only with some devices could you even achieve these consequences if you’re doing it wrong. And that’s why I don’t think it would make sense to put it on all the devices, because it would probably be a waste of money if you don’t really need it in that area.

[GS3]

The interviews have shown that one thing instruments that can apply excessive force to human body tissue have in common is that the force needed to reach the operative goal is specified, but the mechanics of the instrument do not define practitioners’ force application: “With most of the devices that we use for suturing and such things, the pressure is already predefined by the mechanics” (PD1).

Thus, many surgical instruments are designed in a way that prevents the application of excessive forces. Examples of such instruments were electrocautery scissors, forceps, and saws:

But whether you use the saw more or less depends on the saw. You just have to operate it.

[GS1]

### Theme 3: Barriers to Using a Force Sensor in Practice

Despite defining the opportunities to use a novel force sensor across surgical disciplines, participants also discussed factors that could prevent the use of a force sensor inside the operating theater.

#### Concerns About Implementation

Even with acknowledging the potential benefit of real-time feedback on exerted forces, many participants doubted the successful definition of specific safe force ranges to provide them with informative real-time feedback on the force they exert on their patients. Some participants argued that, despite their extensive experience, they do not know what the right or wrong amount of force to be applied is during specific procedures: “Although I have been doing this for many years, I wouldn’t know at what pressure a problem for the bone occurs” (DS4). Furthermore, participants struggled to imagine how such force ranges would be determined while considering all variables that determine appropriate forces: “I would probably find it difficult because there are so many factors that are variable due to the different instruments and different tissues that I can’t standardise” (PD1).

Participants doubted the establishment of safe force ranges for pressure measured by a novel sensor because they reported exerting a complex composite of forces on their instruments. For instance, some participants remarked that they apply pressure with multiple areas on the hand during instrumental work. Others mentioned also applying pulls and torques during procedures with significant physical components:

So, with forceps we are not using the palms of our hands that much really. We’re using more traction and our fingers and actually in reality probably our biceps.

[OG2]

While the componential exertion of force makes it difficult to determine how much force is adequate when measured at the sensor, most participants also raised concerns about the vast inter- and intraindividual differences between patients’ tissues. Most participants feared that these differences in tissue quality would prevent the definition of force ranges calibrated to patient needs:

From patient to patient? They are infinite. So, it’s actually different for everyone. So, also for me the question is really how can you manage that in the end?

[DS2]

Nonetheless, some interviewees mentioned that individual differences could be predicted using advanced imaging methods or accounted for by creating force ranges for standardized patient groups: “If you have a bell curve for different types of people you could sort of establish a range of forces that could potentially show the doctors where it gets critical” (PD3). However, this approach cannot account for intraindividual tissue differences such as the effects of scarring from earlier operations. Consequently, the tissue properties’ unpredictability could limit the feedback’s informativeness. Overall, participants identified several issues that could complicate establishing safe force ranges adapted to the specific procedure and patient.

#### Concerns About Need

Many participants were doubtful about the need for a sensor and the feedback it generates and posited different reasons surgeons might not need a sensor implemented in their work. First, force feedback was voted redundant whenever surgeons could rely on other sources of information to determine how much pressure they should apply. For some participants, their years of clinical experience were sufficient information: “I think once you’ve done a couple of thousand, you don’t necessarily need a quantitative measure because your experience and your sense of feel is used to it” (AN2).

In addition to relying on their experience, several participants explained that, in most procedures, visual or tactile feedback is available at the operating site, making the additional force feedback redundant:

You usually feel a bit of a give and then you see a literal white tissue. When you get to that plane, you don’t go any deeper and then you cut away your specimen.

[PD2]

While sensory information might make force feedback from a novel sensor redundant, participants found scenarios in which any information about their exerted force is unnecessary. Whenever the potential to cause harm is very slight, participants claimed no need for feedback on their exerted forces. This was often the case in coarse procedures where the required force is unconstrained:

In trauma surgery, I think it’s probably more difficult to integrate such a sensor, because there’s a lot of force involved. [...] Theoretically you can’t use too much force.

[GS3]

Although some participants judged the force feedback unnecessary for their work, others questioned the costs relative to the benefits: “I’m not sure how useful this would be only because you’ve got to think whether it’s worth paying for something like that when actually removing something on the abdomen or the leg is a pretty easy procedure in the first place” (PD2).

While these comments referred to preoperative costs, other participants expected adverse operative outcomes. Some participants were worried about the negative impact on their tactile senses from wearing the sensor: “Surgeons work a lot with the tactility of the fingertip and if there’s something in front of it, then that can also be a disadvantage” (GS2). Additionally, some participants were cautious about the impact the interpretation of the real-time feedback would have on their performance: “I would like to be looking at the lesion not at something else to look at pressure” (PD2). Furthermore, experienced surgeons were worried about junior surgeons’ neglect of organic information: “The disadvantage of technology is sometimes that young anaesthetists no longer look at people” (AN1). Further, some medical practitioners who regularly interact with their patients during surgery explained that real-time feedback could stress the patients. Therefore, the interviews revealed a series of procedure- and practitioner-specific factors that limit the value of real-time feedback on surgeons’ intraoperative forces.

### Theme 4: Implementation of a Force Sensor

The final recurrent theme from participant interviews covers participants’ accounts of the implementation of a force sensor. Having been introduced to the concept of a novel force sensor, practitioners frequently discussed how the sensor and the real-time feedback should be implemented to generate the most significant benefit for them.

#### Application of the Sensor

While existing force-sensing systems applied in medicine are predominantly applied to surgical tools, a novel force sensor was developed to be applied to surgical gloves. Many surgeons appreciated the prospective benefits of wearing a sensor on their surgical gloves during instrumental surgery. DS1 proposed implementation of a force sensor on the glove when operators expect to use many instruments in quick succession: “In order to be able to keep up with the speed, I would suggest to rather have it on your finger.” Other proposed advantages of wearing the sensor on the glove were the relative ease of installation and use:

Presumably it would be a bit of faff having a long wire draping out of the syringe, you’re waggling around someone’s back. With Scrubs, it would just reach through your scrub and up your sleeve and down.

[AN2]

Looking ahead, an advanced, wireless force sensor would allow for effortless installation and use at both the tool-tissue interface and the surgeon-tool interface. However, in addition to its implementation during instrument use, many participants suggested the application of the sensor to the glove for manual examinations, particularly during diagnostic maneuvers:

This examination includes an examination of the chewing muscles and also of the temporal lobe up here. And I could also imagine that this pressure sensor would be used to know how hard you have to press.

[DS3]

During instrumental and manual manipulation, surgeons expressed various preferences regarding a sensor’s local application on a glove. Participants’ preference for where a sensor should be applied depended on the instruments used during the respective maneuver. However, different operators also described having personal preferences regarding using certain instruments: “This is a bit specific, because different people will use the syringe in different ways” (AN1). While in most cases, a sensor’s application on a glove was voted preferable by the participants, some interviewees voiced their preference for force measurement at the instrument to know the magnitude of the force exerted at the tool-tissue interface rather than the surgeon-tool interface:

The interesting thing is the pressure that ultimately acts on the tip of the forceps. Not the one I apply, but the one that acts on the tip.

[PD1]

The interviews demonstrated that force measurement conducted at the tool-tissue interface was generally more requested when the forces exerted by the surgeons were complex composites of different hand areas:

I guess then you can then maybe have the sum of the two forces. Whereas if it’s on the finger, you’d have to choose in terms of whether it’s on the thumb or the hand that’s pressing the thing or both.

[OG3]

#### Feedback Modality

Many participants expressed their opinion regarding the feedback modality when considering the reception of real-time feedback during interventions, which were mostly based on a prototype used in the opening video (Multimedia Appendix 1): “But I think if you were to devise an instrument and it had a red bar on it and my force was going towards the red bar or the higher end of the red bar, then I would start paying attention to that” (OG2).

However, most participants who discussed the impact of the visual feedback criticized that the visual feedback could distract surgeons from the operating site: “But a display means that you have to look at it again, and of course you don’t look away when you’re operating” (GS2). Instead, those participants were pleading for visual feedback “where you don’t have to widen your field of vision” (GS3).

Auditory feedback was initially considered to be inappropriate by the developers of the sensor due to the existing audible alarms in the operating theater. This view was confirmed by those participants who reported working in a noisy environment and supported by those working in particularly stressful conditions, where audible feedback was thought to stress the surgeons and the patients:

I spend my life trying to make stressful situations as calm as possible. The last thing I want to do is make it more stressful.

[OG2]

Nonetheless, many interviewees suggested auditory feedback as a viable alternative to visual feedback. The primary benefit of auditory feedback is that it can quickly catch the surgeons’ attention: “The one that we use more is the sound because as soon as it starts to go off the radar with the beeping, you kind of know that that’s the area that you’re interested in” (PD3).

Other participants, who criticized auditory and visual feedback, suggested administering tactile feedback: “I suppose something a bit like the electric toothbrushes, if you’re pressing too hard it will vibrate” (AN2). However, other participants contradicted this again since: “if your fingertips vibrate all of a sudden, it might be strange” (GS3). Altogether, the interviews revealed that surgeons must choose whatever feedback works best for them, depending on which senses are occupied by environmental stimuli and which are available.

## Discussion

### Principal Findings

Our research has shown how a novel force sensor could be valuable in clinical practice, specifically at the surgeon-tool interface. All conclusions are formative, as they reflect perspectives from a small but highly relevant sample and do not account for the full complexity of real-world surgical scenarios. In total, four themes with specific implications were distilled based on their relevance to the research objectives (Table 2). In particular, the findings propose the application of the sensor when performing procedures where other sources of information guiding force exertion are masked, manipulating tissues that are susceptible to excessive force due to their importance for bodily functioning, or using instruments that are prone to cause harm to the tissue due to their mechanical design. However, participants questioned the value of the sensor when performing maneuvers where individual tissue differences are significant, force compositions are complex, or there is enough explicit and tacit knowledge on which surgeons can base their force judgment. Finally, the findings highlighted the need for domain-specific determination of appropriate places to apply the sensor and an appropriate feedback modality.

**Table 2.** Key implications of the present findings.

Themes	Implications for application
Theme 1: Using a novel force sensor could ...	Help practitioners apply adequate force magnitudes during surgery Provide objective surgical skill training and quantifiable assessment
Theme 2: A novel sensor should be applied for ...	Maneuvers where other sources of information guiding force exertion are masked Tissues susceptible to excessive force due to their importance for bodily functioning Instruments prone to cause harm to the tissue due to their mechanical design
Theme 3: A novel sensor should not be applied in ...	Procedures with large individual tissue differences and complex force compositions Procedures with enough explicit and tacit knowledge to base force judgment on
Theme 4: A novel sensor could be implemented ...	At various hand areas to measure force during instrumental and manual procedures At the instrument when the force is a complex composite from multiple hand areas With feedback depending on the accessibility of the visual, auditory, and tactile senses

### Limitations

This study explored 15 surgeons’ views on the sensor glove’s usability. As a formative study, the sample size aimed to ensure comprehensiveness across relevant contexts while avoiding placing excessive demands on busy health professionals. While these participants came from various medical specialties to ensure a balance between representing a broad range of medical specialties and allowing for aggregation of interview results, the sample does not represent the entire surgeon population or all surgical specialties across the medical landscape. Thus, the results presented here might not generalize to the perspectives of practitioners from other specialties. By conducting this study’s interviews primarily online, there was reduced rapport between the interviewer and the interviewee, which could have reduced participant engagement.

Finally, the interviewer’s lack of medical training could have adversely affected the interviews, though they took steps to familiarize themselves with the specifics of the different procedures performed by participants; conversely, the interviewer was not involved in the development of the sensorized glove and was not biased by being trained in one of the medical specialisms included in the study, thereby removing many other sources of bias.

Participants noted ethical and ergonomic challenges such as sterilization, tactile interference, and data privacy, but these fall outside the scope of this formative study and will be addressed in future design and validation phases.

### Comparison With Prior Work

#### Benefit of Using the Sensor

Using the sensor could benefit surgeons in two ways: by enhancing the operative outcomes of interventions and improving the training of junior surgeons. We found that participants expected that wearing the sensor during surgery could enhance operative outcomes, reinforcing the notion that real-time feedback on exerted forces can improve the quality of surgery [1,18,35]. Nonetheless, like Ebrahimi et al [35], most existing research has only assessed the impact of feedback on the exertion of intraoperative force measured at the tool-tissue interface. Our findings extend this knowledge by suggesting that feedback on exerted forces could also enhance operative outcomes when measured outside the patient’s body at the surgeon-tool interface.

Most participants agreed that the sensor could help junior surgeons develop a feeling for adequate intraoperative forces, overcoming the difficulties of learning from qualitative feedback described in the scientific literature [2,22]. We found that a novel force sensor could be used for training purposes both in and out of the operating theater. This supports Rhienmora et al [36], who suggested that the feedback serves to develop a feel for adequate forces and can be interpreted for assessment purposes. Thus, the sensor could accelerate surgical skill acquisition through objective feedback in real time and serve as a novel quantifiable metric.

#### Opportunities for Using a Force Sensor in Practice

First, our results revealed that surgeons should use a force sensor when performing maneuvers where other sources of information on intraoperative forces are masked. These results corroborate existing studies that have attributed poor surgical outcomes to errors of perception rather than poor motor skills [37,38] and initially inspired researchers to test the provision of feedback on exerted forces during surgery [39]. Because organic force feedback is often masked in standard open surgeries, we propose that feedback on exerted forces from a novel sensor should be provided in those scenarios.

Second, our results demonstrated that a force sensor should be applied when tissues are susceptible to inappropriate force due to their relative importance for bodily functioning. This finding supports previous research that has highlighted the importance of force sensing when surgeons deal with delicate structures in retinal or nervous tissue, as they require minimal force [11,12,25]. Our findings extend this view, as they propose that a particular tissue’s importance for bodily functioning does not just depend on its delicacy but also on that tissue’s healing qualities. The current results demonstrate that a sensor could also have high applicability when dealing with more coarse tissue with poor healing abilities, such as bone tissue.

The application of a novel sensor when dealing with instruments prone to cause harm to the patient was also identified as an opportunity, as previous research has indicated that the design of interactive medical devices can provoke user error [40]. Our study supports these findings since it revealed that where the exertion of intraoperative force is not predefined by the instrument’s mechanics, the chance to harm patient tissue is high (in contrast to instruments where the mechanics define the exertion of force). Although our interviews revealed some examples of the latter (eg, electrocautery scissors, forceps, and saws), this has not been reported in prior literature.

#### Barriers to Using a Force Sensor in Practice

The implementation of accurate force ranges is challenged by vast individual differences between patients’ tissues. This finding is supported by previous studies that identified significant differences in tissue qualities between individuals [26] and within individuals [27]. Building on the findings by Jacquet et al [41], which demonstrate that significant variability in skin mechanical properties persists even after controlling for sex, age, and BMI, our findings suggest that fixed force ranges for real-time feedback may be insufficiently informative for patients whose tissue qualities deviate from the norm. Thus, the sensor should be applied in cases where inter- and intraindividual differences are minor or predictable.

Additionally, implementing force ranges is challenged by the multimodality of the forces that surgeons use to manipulate their patients. Apart from the linear manual pressure applied on the fingertip, which the sensor can achieve, intraoperative forces can be complemented by torques or pulls, which are activated by different parts of the surgeon’s body, such as the wrist or the biceps [42]. Therefore, this sensor should only be applied in scenarios where the exertion of intraoperative force is less complex.

Finally, surgeons’ ability to rely on their experience and other information sources challenges the need for real-time feedback on exerted forces to be displayed. Our findings support existing conceptions of direct visual and tactile feedback’s impact on the operator’s judgment [43]. During surgical manipulation of human tissue, visuotactile neurons in the human brain allow surgeons to combine visual and haptic information into one judgment [44]. Our study supports the existing conception that surgeons judge their exerted force by relying on their experience and that surgical clinical judgment necessitates decisions blending explicit knowledge derived from evidence with tacit knowledge drawn from experience [45]. Thus, the sensor might be redundant when surgeons have enough explicit and tacit knowledge to base their judgment on. However, in high-risk situations or low-visibility scenarios, the sensor may still be of use to experienced surgeons.

#### Implementation of a Force Sensor

Although participants did not interact with a physical prototype, their feedback highlights key design considerations for future iterations and provides an initial framework for usability testing in subsequent studies. Importantly, these design considerations need to be adapted for different surgical specialties. For example, dentists may require sensor placement optimized for fine instrument handling, whereas obstetricians may need configurations suited for manual examinations. Opie et al [46] explored the use of a sensorized glove in a usability study to assist with vaginal examinations during childbirth. It consisted of 18 participants either specializing in childbirth or with the intent to work in women’s health. That study involved creating a realistic environment where the usability of the device could be assessed in a safe and effective manner. Participant feedback from that study had a direct influence on the design of the sensorized glove for future iterations.

We identified that a novel force sensor and the associated real-time feedback should be implemented in multiple areas of the hand depending on the instrument. Additionally, by measuring intraoperative force at the surgeon-tool interface, a novel force sensor could also be used for manual treatments and diagnostic procedures to improve surgeons’ application of adequate forces during manual procedures as previously suggested in physiotherapy where they showed a temporary performance improvement when provided with force feedback [47,48].

When the force exerted onto tissue is a complex composite of forces applied by the hand, the sensor should be at the instrument tip. Aggravi et al [29] measured the contact forces exerted on three surgical tools and discovered a significant difference in forces applied by the thumb and index finger. Although these findings imply that a glove could have multiple force sensors, interpreting the different feedback strands could be challenging. This was supported by our participants, who suggested that measuring force at the tooltip to receive information on the sum of the force applied by different hand parts was more practical.

Finally, the indirect force feedback modality should depend on the accessibility of the visual, auditory, and tactile senses. Previous research discussed the benefits and drawbacks of providing visual, auditory, and tactile feedback in clinical settings [49-52], leading to the conclusion that the choice of feedback modality cannot be generalized. These findings were confirmed by a recent survey among health care professionals who stressed the importance of predetermining the best procedure-specific feedback modality based on the load on different senses in the operating environment [53]. Ultimately, it might come down to creating custom configurations of more than one feedback modality, as introduced by Tajadura-Jiménez et al [54], who introduced multisensory integration processes used to alter the perceived materiality of a touched surface to shape people’s touch behavior.

### Conclusions

This study explored the usability of a conceptual novel sensor for measuring intraoperative force at the surgeon-tool interface. Insights were gathered from semistructured interviews with 15 surgeons from different medical disciplines and experience levels who evaluated the concept of a force sensor provided by a video showcasing a conceptual force sensor. This formative study found that wearing a force sensor could enhance surgical outcomes by aiding surgeons in applying appropriate force levels and facilitate training for aspiring surgeons by providing an objective metric of force. Practical applications of the new sensor include maneuvers with little feedback on exerted forces from organic sources, where the tissues manipulated are vital for bodily functioning and have poor healing qualities, and where the instruments used are prone to inflict harm because of their mechanical design. However, the sensor’s usability is limited, where establishing safe force ranges is complicated by significant individual differences in tissue qualities and complex force compositions, and where experienced surgeons do not need the sensor due to sufficient explicit and tacit knowledge of their exerted force. The implementation of the sensor at various hand locations is feasible. However, its placement on the instrument is preferred when exerted forces are complex composites from multiple areas on the hand. Finally, the feedback modality should be tailored to specific maneuvers. While substantial benefits, opportunities, and potential for using a sensor at the surgeon-tool interface exist, future research should address barriers and optimize its implementation to realize its benefits.

## Supplementary material

10.2196/78845 Multimedia Appendix 1 Video used in the study to introduce an exemplar novel force sensor.

10.2196/78845 Multimedia Appendix 2 The semistructured interview guide used in the study.

## Abbreviations

REDCap: Research Electronic Data Capture
