# Supplementary material for: Surgeons’ Perceptions on the Utility of a Conceptual Novel Force Sensor at the Surgeon-Tool Interface: Formative Interview Study
Source: JMIR Form Res. 2026 Apr 27;10:e78845. doi: 10.2196/78845 (PMC13120544; doi:10.2196/78845)
Supplement: Multimedia Appendix 2 [file formative-v10-e78845-s002.pdf]

# Semi-structured Interview Guide

1. **Introduction**
   - Welcome and thank you for making time to take part in this study.
   - The aim of today's session is to understand how a novel force-sensing surgical glove could enhance the outcomes of surgery.
   - Research has suggested that feedback of surgeons' exerted force could help apply optimal force, reducing the risk of complications and improving patient outcomes.
   - A new sensor measures the force which surgeons exert onto their instruments or the patient, allowing real-time feedback of the exerted force to be displayed to surgeons.
   - Now, it must be qualitatively explored where different surgical practices could benefit from real-time feedback of the exerted force from the sensorised glove.
   - With your help, we aim to identify scenarios within *perinatal medicine* for which the sensor glove could enhance surgery outcomes.
2. **Informed consent**
   - Provide information sheet and consent form, and ask participant to read and sign it
   - Do you have any questions regarding the research study ahead?
3. **Introduction to the sensor glove**
   - Show Video
   - Do you have any questions regarding the functionality or the use of the sensor glove?
4. **Semi-structured interview using journey map**
   - *Introduction to interview*
     - As mentioned, we aim to identify scenarios in which receiving real-time force measurement from the sensor glove could enhance surgery outcomes.
     - Our goal is to generalise these scenarios across different surgical disciplines.
     - I would like to go through the surgery that you provided as a representation of your daily work and discuss its different steps in terms of some factors that might influence the applicability of the sensor glove in that specific procedure.
     - Specifically, I will ask you about the instruments you use, the types of human body tissue you are manipulating, and the actions you are performing
     - I will also ask you to evaluate if receiving real-time force measurements from the sensor glove could improve the safety of the specific manoeuvre."
     - Do you have any questions at this stage? Otherwise, I would suggest we begin with the interview, and I start the recording.
   - *Warm-up questions*
     - To start, could you tell me a little bit about your surgical activities, in terms of the practice you work in and the number of years of experience you have.
     - You sent me information on the procedure of *forceps delivery*. How often do you perform this procedure?
     - Do you face any challenges during this surgery?
       - Are there any challenges that concern the safety of the patients?
       - Do you see errors in force application as a source of these challenges?
     - Force sensing mechanisms have been introduced in some types of surgery in recent years. Have you used force-sensing mechanisms during surgery before?
       - If yes/no, what are your thoughts on current methods of force sensing?
     - A big factor in force sensing is the display of real-time feedback of the exerted force. Have you previously received real-time feedback of the exerted force during surgery?
       - If yes, how did the feedback of the exerted force impact your surgery?
       - If no, have you thought about the potentials of feedback of the exerted force before?

- *Interview: Repeat for each of the surgical steps along the journey map*
  - I have tried to simplify the information you sent me into this journey map. The journey map will serve as a reference point to discuss the individual surgery steps and their potential for benefiting from feedback of the exerted force
  - Step 1: *Emptying the Bladder*
    - What instruments do you use to perform this step? How do they work?
      - How important is applying the right force to these instruments?
        - Why is it (not) important?
        - Are there any indications that too much / too little force is applied?
        - What happens if you apply too much or too little force?
        - What impact does this have on patient outcomes?
    - What type of human body tissue are you manipulating?
      - How important is applying the right force to this tissue?
        - Why is it (not) important?
        - Are there any indications that too much / too little force is applied?
        - What happens if you apply too much or too little force?
        - What impact does this have on patient outcomes?
    - What action are you performing to manipulate the tissue?
      - How important is applying the right force during this action?
        - Why is it (not) important?
        - Are there any indications that too much / too little force is applied?
        - What happens if you apply too much or too little force?
        - What impact does this have on patient outcomes?
    - Have you heard of any instances where the wrong force was applied during this manoeuvre?
      - What complications arose because of that?
      - What was the impact on patient outcomes?
      - What else could have happened if the wrong force had been applied: too much vs too little pressure?
    - **Do you think, receiving real-time force measurements from using the sensor glove could improve the safety of this specific scenario?**
      - What would this potential benefit look like?
      - If yes, how would this look like in the workflow?
        - Glove switching, instrument switching etc.
- *Closing questions (if time is left)*
  - "One way in which the surgical glove could be applied outside the operating theatre is in the training and assessment of surgical trainees. What are the most critical scenarios in which the surgical glove would be particularly beneficial for training and assessment of surgical trainees?"
    - "Why?"

**4. Ending the session**

- Is there anything else you would like to add that you have not been able to discuss yet?
- We have reached the end of the interview. Thank you. Do you have any questions?
